# *MicroRNA-587* Functions as a Tumor Suppressor in Hepatocellular Carcinoma by Targeting Ribosomal Protein SA

**DOI:** 10.1155/2020/3280530

**Published:** 2020-09-05

**Authors:** Miao Chen, Duo Wang, Junjie Liu, Zhizhan Zhou, Zhanling Ding, Lianfeng Liu, Danke Su, Hang Li

**Affiliations:** ^1^Centre of Imaging Diagnosis, Guangxi Medical University Cancer Hospital, Nanning, Guangxi 530021, China; ^2^Departments of Ultrasound, Guangxi Medical University Cancer Hospital, Nanning, Guangxi 530021, China

## Abstract

**Background:**

Hepatocellular carcinoma (HCC) is one of the most highly aggressive cancer worldwide with an extremely poor prognosis. Evidence has revealed that *microRNA-587* (*miR-587*) is abnormally expressed in a series of cancers. However, its expressions and functions in HCC have not been clearly acknowledged.

**Methods:**

We detected the expression level of *miR-587* both in the Gene Expression Omnibus (GEO) database and 86 paired clinical HCC tissues together with paired adjacent normal tissues by quantitative real-time PCR (qRT-PCR). Afterwards, the transfected HCC cell line SMMC-7721 cells were collected for the cell proliferation assay, cell-cycle arrest, cell migration, and invasion assays to explore the roles of *miR-587* in regulating cellular function. In addition, bioinformatics analysis, combined with qRT-PCR and dual-luciferase reporter assays, were performed to confirm whether ribosomal protein SA (*RPSA*) mRNA was the direct target gene of *miR-587*. Moreover, the Cancer Genome Atlas (TCGA) and GEO databases as well as 86 paired clinical HCC tissues were used to verify the negative regulation between *miR-587* and *RPSA*.

**Results:**

In the present study, both the GEO database (GSE36915 and GSE74618) analysis and qRT-PCR analysis of 86 paired clinical tissues showed that *miR-587* was significantly downregulated in HCC tissues. The overexpression of *miR-587* inhibited proliferation, cell cycle, migration, and invasion in SMMC-7721 cells. In addition, miR-587 directly interacted with the 3′-untranslated region (UTR) of *RPSA*. Moreover, *miR-587* overexpression directly suppressed *RPSA* expression, and the two genes were inversely expressed in HCC based on the analyses in TCGA and GEO (GSE36376) databases and qPCR analysis of 86 paired clinical tissues.

**Conclusion:**

Our results demonstrate that *miR-587* is downexpressed in HCC and regulates the cellular function by targeting *RPSA*.

## 1. Introduction

Primary liver cancer is the fourth leading cause of cancer-related death worldwide, and the incidence rates continue to rise faster than that of any other cancer in both men and women [1]. HCC accounts for 85–90% of all cases of primary liver cancer, which poses a significant threat to health and life in China [2]. MicroRNAs (miRNAs) are a set of small, noncoding RNAs of 20-25 nucleotides which play a pivotal role in regulating gene expressions by directly targeting the 3′-UTR of mRNAs and negatively regulating its transcription and translation [3]. Growing evidences reveal that miRNAs exert critical functions in the progression of multiple human cancers [4, 5]. As to HCC, numerous studies have reported that up- and downregulations of miRNAs are closely related to the occurrence and prognosis of HCC [6, 7]. For instance, Ji et al. [8] report that the upregulation of *hsa-miR-210* can predict the poor outcome of HCC. Xiang et al. [9] show that downregulated *miR-520d-3p* is correlated with the poor survival of HCC patients, which may attribute to its promotion on cell proliferation, migration, and invasion in HCC cells. Wei et al. [10] conclude that *miR-137* is correlated with the poor prognosis of HCC patients based on the microarray data of GSE31384. Related studies show that *miR-587* can be abnormally expressed in colorectal cancer [11], melanoma [12], and glioma [13]. However, its expressions and cellular functions in HCC have not been fully elucidated. In this study, we aim to systematically investigate the expression levels and biofunctions of *miR-587* in HCC.

*RPSA*, a laminin receptor 1, is a member of the nonintegrin family. The amino acid sequence of RPSA is highly conserved during evolution and has important biological functions by interactions with the extracellular matrix glycoprotein laminin [14, 15]. Munien et al. [16] report that RPSA transcript levels are higher in malignant melanoma cells than normal cells. However, the expression and biofunction of RPSA in HCC are not fully explored.

In this study, we analyzed the relative expressions of *miR-587* in HCC tissues and adjacent normal tissues and explored its cellular functions in SMMC-7721 cells. Furthermore, whether *RPSA* was a target mRNA of *miR-587* was predicted and verified, and their regulatory relationship in HCC was analyzed.

## 2. Materials and Methods

### 2.1. Tissues and Patients

Eighty-six cancerous tissues and adjacent normal tissues from patients pathologically diagnosed as HCC from April 2013 to September 2016 at the Affiliated Tumor Hospital of Guangxi Medical University, China, were sampled. The tissues were stored at -80°C in RNAlater (Qiagen, Valencia, CA, USA). All patients enrolled met the criteria that were defined as follows: (a) patients undergoing surgical resection initially, (b) who were diagnosed according to histopathological criteria, and (c) without any other malignant tumors. Clinical characteristics consisted of age, sex, smoking status, alcohol use, tumor size, HBV infection, number of involved lesions, serum alpha-fetoprotein (AFP) level, liver cirrhosis, ascites, the Barcelona Clinic Liver Cancer (BCLC) stage, TNM stage, tumor thrombus, distant metastasis, microvascular thrombi (MVI), and early postoperative recurrence (≤12months). All data were obtained from medical records and pathological reports of the patients. The ethical approval for our research on human HCC tissues after surgical resection was obtained from the Ethics Review Committee of the Affiliated Tumor Hospital of Guangxi Medical University.

### 2.2. Cell Lines and Culture Conditions

The liver cancer cell line SMMC-7721 cells were preserved in our laboratory and identified by short tandem repeat (STR) analysis (GENEWIZ Inc., South Plainfield, NJ, USA). Cells were cultured in Dulbecco's modified Eagle medium (DMEM, Thermo Fisher Scientific, USA) supplemented with 10% fetal bovine serum (FBS, Gibco, Grand Island, USA) and 1% antibiotics (penicillin, 100 *μ*/ml; streptomycin sulfates, 100 mg/ml) in a humidified atmosphere with 5% CO_2_ at 37°C.

### 2.3. RNA Extraction, Reverse Transcription, and Quantitative Real-Time PCR

TRIzol reagent (Invitrogen, Carlsbad, CA, USA) was used to extract total RNA from the enrolled tissues and cultured cells. The concentration of total RNA was determined using a NanoDrop 8000 spectrophotometer (Thermo Fisher Scientific, Waltham, MA, USA). Sepharose was used to bind the integrality of RNA. Then total RNAs in tissues and cells were reverse transcribed into cDNA using the miRcute miRNA cDNA kit (Tiangen Biotechnology, Beijing, China) and PrimeScript™ RT reagent kit with gDNA Eraser (Takara, Dalian, China). Additionally, a qRT-PCR platform (Roche Lightcycler 480 system, Roche Diagnostics, Basel, Switzerland) was employed to determine expression levels of *miR-587* and *RPSA* using the miRcute miRNA qPCR detection kit (Tiangen Biotechnology, Beijing, China) and FastStart Universal SYBR Green Master (ROX) kit (Roche, Germany). Relative expression levels of *miR-587* and *RPSA* were normalized to their endogenous controls *U6* and *Actin beta* using the 2^−*ΔΔ*Ct^ method.

### 2.4. Cell Transfection

Synthesized RNA duplexes of *miR-587* mimics, *miR-587* inhibitors, and conjugated negative controls (NC) were purchased from Tiangen Biochemical Technology (Beijing, China). According to the manufacturer's instructions, cells (1 × 10^5^ per well) were seeded into 12-well plates. *miR-587* mimic and mimic NC (both at the concentration of 50 nM) as well as *miR-587* inhibitor and inhibitor NC (100 nM) were added to cells at the logarithmic phase of growth. Lipo6000 (Invitrogen, USA; 5 *μ*l per well) was used for transfection per well as well. The cells were harvested after 48 h of transfection to determine the relative expressions and analyze altered functions of *miR-587*.

### 2.5. Cell Proliferation Assay

The 3-(4,5-dimethylthiazol-2-yl)-2,5-diphenyl tetrazolium bromide (MTT) assay was performed to detect the proliferation of SMMC-7721 cells. SMMC-7721 cells (approximately 5000 per well) transfected by mimics or inhibitors were collected and seeded into 96-well plates, and their viability at 24, 48, 72, and 96 h was measured. The cells were incubated with 100 *μ*l culture medium mixed with 10 *μ*l MTT (Sigma-Aldrich, St. Louis, USA) solution for 4 h. And then, the mixed medium was replaced by dimethylsulfoxide (DMSO, Sigma-Aldrich; Shanghai, China), 150 *μ*l per well. Subsequently, the plate was agitated on the shaking table (TSB-108, Qilinbeier, Jiangsu, China) for about 15 min in the dark. The optical density (OD) value at 490 nm was quantitated for each sample to obtain the viability of SMMC-7721 cells.

### 2.6. Cell Wound Healing Assay

SMCC-7721 cells (1 × 10^5^ per well) were seeded into 6-well plates. After adherence, the cells were transfected with *miR-587* mimic and inhibitor as well as the corresponding NC for 48 h, respectively. A wound (a clear straight line) was vertically scraped across each well using a sterile 200 *μ*l pipette tip. Phosphate buffered saline (PBS; Gibco) was used to clean the floating cells. Subsequently, the 6-well plates were cultured with serum-free DMEM. The cells were photographed after 0 and 24 h of incubation using a digital camera system (Olympus Corporation, Tokyo, Japan).

### 2.7. Transwell Invasion Assay

Transwell assay was carried out to detect the cell invasion in 24-well plates (BD Bioscience, USA) with Transwell filters of 8 *μ*m pore size (Costar, USA). Transfected cells (1 × 10^5^ per well) cultured with serum-free DMEM were added to the upper chamber. The lower chamber was filled with DMEM containing 10% serum. The cells were allowed to invade for 24 h, and those on the lower side of Matrigel filter were fixed with paraformaldehyde, stained with crystal violet, and photographed and counted using a microscope.

### 2.8. Measurement of Cell Cycle

After 48 h of transfection, about 1 × 10^6^ cells were collected and fixed in 70% ethanol overnight at -20°C and then washed three times by PBS. The cells were added with 0.25 mg/ml RNase A for incubation at 37°C for 30 min. And 5 *μ*l propidium iodide (PI, KeyGen, Nanjing, China) was further added for 30 min incubation at room temperature in the dark. Cell suspension was collected for analyzing cell cycle using the FACSCalibur Flow Cytometer (BD Bioscience, USA).

### 2.9. miR-587 Target Prediction

The TargetScan Release 7.1 prediction algorithm was used to predict the potential target genes of *miR-587* (http://www.targetscan.org/vert_71/). The cumulative weighted context++ score was utilized to screen the putative *miR-587* targets. Genes with a cutoff value of less than -0.4 were considered as the potential targets. Meanwhile, the significantly upregulated genes in HCC were collected in TCGA databases. The final targets were confirmed based on the interaction between the predicted *miR-587* targets and the upregulated genes in HCC.

### 2.10. Dual-Luciferase Reporter Assay

A miR-RPSA-3′-UTR plasmid containing the potential *miR-587* binding sites was prepared by using the Dharma FECT Duo Transfection Reagent for the human RPSA-3′-UTR luciferase reporter assay. 293T cells were cotransfected with the luciferase plasmid (100 ng per well) and *miR-587* mimics (100 nM) in 96-well plates. After the cells were transfected for 48 h at 37°C, the luciferase activity was detected using the dual-luciferase reporter assay system (Promega, Madison, USA).

## 3. Statistical Analyses

All statistical analyses in this study were performed using the SPSS 22.0 software (Chicago, IL, USA). Graphs were generated using the GraphPad Prism 5 software (La Jolla, CA, USA). The differential gene analysis of multiplatform transcriptional expression data in GEO and TCGA was performed using the “limma” package in R language. Gene expression profiling datasets of *miR-587* and *RPSA* were examined based on the two databases using Student's *t*-test. Additionally, a paired *t*-test was performed to analyze the relative expressions of *miR-587* and *RPSA* in clinical HCC tissues compared with adjacent normal tissues. Qualitative variables were compared using chi-square tests. Mean ± standard error of the mean (SEM) was presented to describe continuous variables. Pearson's correlation analysis was used to analyze the correlation between *miR-587* and *RPSA* expressions. A two-sided *p* value of less than 0.05 was set as the threshold for statistical significance.

## 4. Results

### 4.1. miR-587 Was Downregulated in HCC Tissues

**Figure 1:**
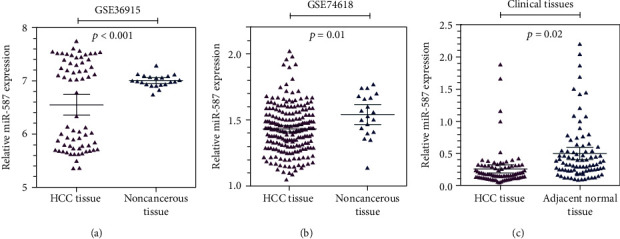
Downexpression levels of *miR-587* in HCC. The gene expression analysis based on (a) GSE36915, (b) GSE74618 datasets from GEO database, and (c) data from 86 paired clinic HCC tissues shows that *miR-587* was downexpressed in HCC tissues compared with noncancerous tissues (*p* < 0.001, *p* = 0.01, and *p* = 0.02, respectively). miR: microRNA.

Two GEO (GSE36915 and GSE74618) databases were included for the validation of *miR-587* expressions. Consistently, the GSE36915 study containing 68 HCC tissues and 21 noncancerous tissues validated that *miR-587* was significantly downregulated in HCC tissues compared with noncancerous tissues (*p* < 0.001, Figure 1(a)). And GSE74618 containing 218 HCC tissues and 20 noncancerous tissues revealed the same result that HCC tissues had lower *miR-587* expression levels than noncancerous tissues (*p* = 0.01, Figure 1(b)). To confirm the results according to bioinformatics analysis from the two databases, 86 paired HCC and adjacent normal tissues were collected in this study. The qRT-PCR analysis showed the same result that expression levels of *miR-587* were significantly reduced in HCC tissues compared with adjacent normal tissues (*p* = 0.02, Figure 1(c)). The relationship between *miR-587* expression and clinicopathologic features of HCC patients is displayed in Table 1. The results showed that *miR-587* was associated with AFP (*p* = 0.002) and MVI (*p* = 0.041).

### 4.2. miR-587 Inhibited Cell Proliferation in HCC Cells

**Figure 2:**
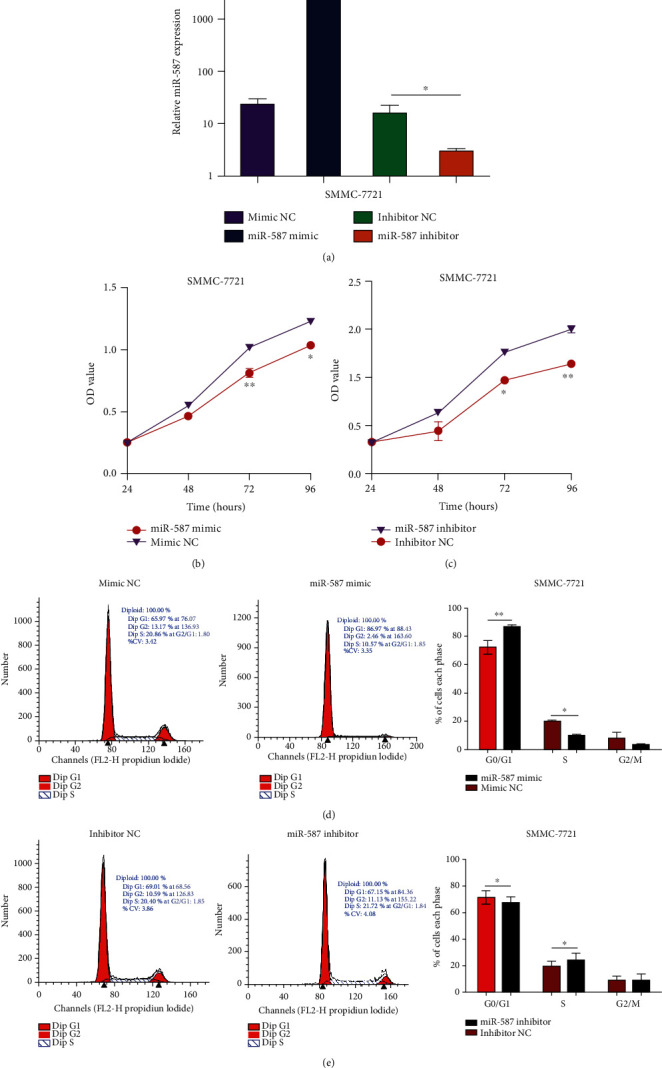
*miR-587* suppressed HCC cell proliferation. (a) The transfection efficiency of *miR-587* mimic (*p* = 0.001) and inhibitor (*p* = 0.037). The MTT assay showed that *miR-587* upregulation (b) suppressed the cell viability of SMMC-7721 cells, whereas *miR-587* downregulation (c) fostered the cell viability (both *p* < 0.05). Flow cytometry analysis showed that the number of SMMC-7721 cells at G1 phase increased and those at S phase decreased in the *miR-587* mimic group (d) (*p* = 0.001, *p* = 0.044, respectively). The number of SMMC-7721 cells at G1 phase decreased and those at S phase increased in the *miR-587* inhibitor group (e) (*p* = 0.028, *p* = 0.048, respectively). ∗*p* < 0.05, ∗∗*p* < 0.01. miR: microRNA.

QRT-PCR assay was used to detect the transfection efficiency of SMMC-7721 cells, and results showed that the relative expression level of *miR-587* in cells transfected with the *miR-587* mimic was 237.94 times higher than that in cells transfected with mimic NC (*p* = 0.001), and *miR-587* expressions in cells transfected with the *miR-587* inhibitor decreased by 18.77% compared with those in cells transfected with inhibitor NC (*p* = 0.037) (Figure 2(a)). Then, the MTT assay was conducted to detect the viability of SMMC-7721 cells. The results revealed that the upregulation of *miR-587* suppressed cell proliferation at 72 (*p* = 0.008) and 96 h (*p* = 0.019) (Figure 2(b)). Conversely, the downregulation of *miR-587* fostered cell proliferation at 72 (*p* = 0.037) and 96 h (*p* = 0.001) (Figure 2(c)). The results of the MTT assay revealed that the overexpression of *miR-587* evidently suppressed the proliferation of SMMC-7721 cells.

Moreover, we explored changes in the cell cycle in *miR-587*-transfected SMMC-7721 cells by flow cytometry. The results showed that in comparison with NC, the number of SMMC-7721 cells significantly increased in the G1 phase of the cell cycle in the *miR-587-*overexpression group (*p* = 0.001), while the number markedly decreased in the S phase (*p* = 0.044) (Figure 2(d)). Besides, the number of SMMC-7721 cells significantly decreased in the G1 phase (*p* = 0.028) and increased in the S phase (*p* = 0.048) in the *miR-587-*downexpression group compared with the NC (Figure 2(e)). These results indicated that the overexpression of *miR-587* induced cell-cycle arrest in the G1 phase and blocked cell division in SMMC-7721 cells after 48 h of transfection.

### 4.3. miR-587 Suppressed Cell Mobility and Invasion in HCC Cells

**Figure 3:**
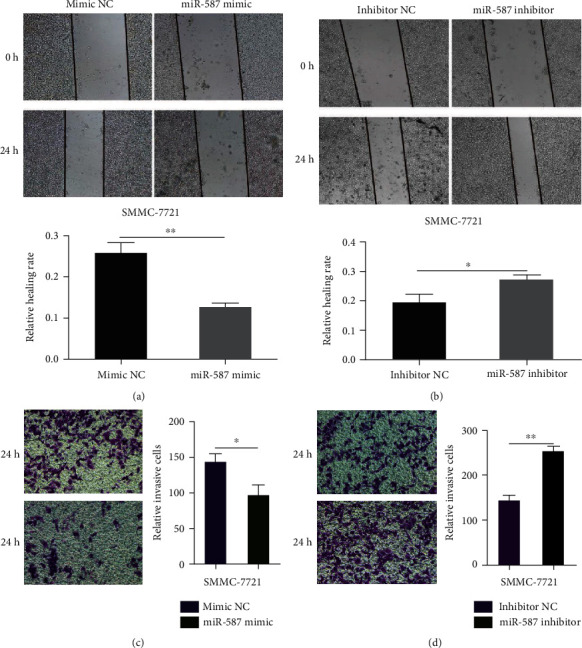
*miR-587* curbed cell migration and invasion in HCC cells. The wound healing migration assay showed that *miR-587* upregulation (a) depressed the migration rate of SMMC-7721 cells and *miR-587* downregulation (b) elevated the migration rate of SMMC-7721 cells (*p* < 0.001, *p* = 0.033, respectively). The Transwell assay showed that *miR-587* upregulation (c) suppressed the invasion and *miR-587* downregulation (d) enhanced cell invasion of SMMC-7721 cells (*p* = 0.034, *p* < 0.001, respectively). ∗*p* < 0.05, ∗∗*p* < 0.01. miR: microRNA.

The wound healing migration assay showed that the migration rate of SMMC-7721 cells decreased in the *miR-587* mimic group at 24 h compared with the mimic NC group (*p* < 0.001) (Figure 3(a)), and the rate increased in the *miR-587* inhibitor group compared with the inhibitor NC group (*p* = 0.033) (Figure 3(b)). The Transwell assay showed that the overexpression of *miR-587* curbed cell invasion—the number of cells on the lower side of the filter decreased—in the *miR-587* mimic group compared with the mimic NC group (*p* = 0.034) (Figure 3(c)). In contrast, cell invasion in SMMC-7721 cells was triggered (or cells on the lower side of the filter increased) in the *miR-587* inhibitor group compared with the inhibitor NC group (*p* < 0.001) (Figure 3(d)).

### 4.4. Bioinformatics Prediction Pinpointed RPSA as the Functional Target of miR-587 in HCC

**Figure 4:**
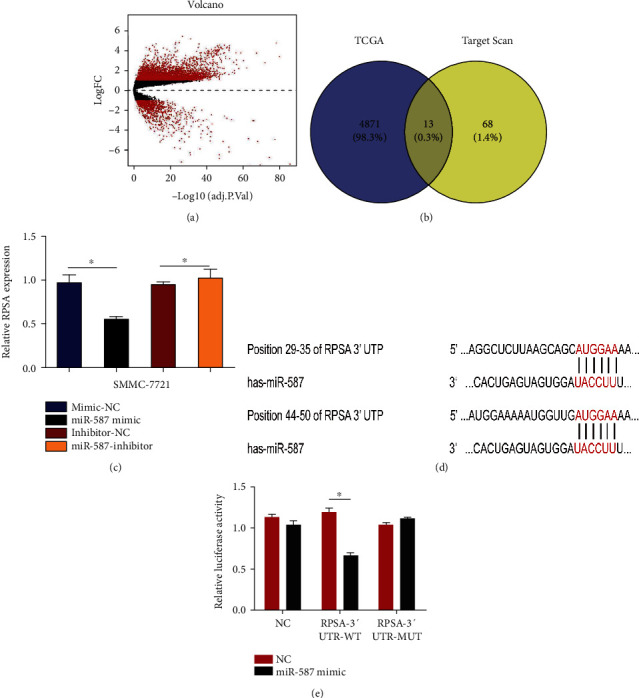
*RPSA* as a direct target of *miR-587*. (a) A total of 4884 overexpressed genes in HCC were screened out based on the TCGA database. (b) Thirteen potential target genes of *miR-587* were included after intersecting outputs between TCGA database and TargetScan. (c) QRT-PCR assay revealed inverse expressions of RPSA and *miR-587* in SMMC-7721 cells in both the mimic group (*p* = 0.014) and the inhibitor group (*p* = 0.024). (d) The 3′-UTR region of RPSA had two target sites interacting with *miR-587*. (e) Dual-luciferase reporter assay showed a specific interaction between *miR-587* and the 3′-UTR of *RPSA* mRNA (*p* < 0.001). ∗*p* < 0.05, ∗∗*p* < 0.01. miR: microRNA.

The TargetScan Release 7.1 prediction algorithm was employed to explore potential *miR-587* targets. The prediction algorithm screened out 81 target genes at the threshold of -0.4. As our results showed a downregulation of *miR-587* in HCC tissues, significantly upregulated genes in HCC were selected based on the negative associations between *miR-587* and its target genes. The analysis of TCGA data revealed that 4884 genes were upregulated in HCC (logFC = 1) (Figure 4(a)). Furthermore, we integrated the predicted target genes of *miR-587* and the upregulated genes in HCC, and results showed that 13 differentially expressed target genes of *miR-587* were found in HCC including *OSR2*, *RAD54B*, *FAM81A*, *DKKL1*, *PTHLH*, *RPSA*, *ZNF781*, *ZNF92*, *CD163L1*, *ZNF138*, *PTPRR*, *TXNL4A*, and *ZNF124* (Figure 4(b)). Followed by literature retrieval in the National Center of Biotechnology Information (NCBI) database, only the gene *RPSA* in the intersection was eligible because it had been reported to be involved in the progression of several cancers, playing a vital role in the proliferation, invasion, and migration in cancer cells [16–18] and showing the inverse cellular function as *miR-587*.

### 4.5. The 3′-UTR of RPSA Was Directly Targeted by miR-587

Furthermore, we used qRT-PCR experiment to verify whether *RPSA* expression was regulated by *miR-587*, and it turned out that cells transfected with the *miR-587* mimic showed a loss of 52.11% of the relative expression level of *RPSA* compared with those transfected with mimic NC (*p* = 0.014), and the *RPSA* level in cells transfected with the *miR-587* inhibitor was 1.172 times higher than that in cells transfected with inhibitor NC (*p* = 0.024) (Figure 4(c)). Subsequently, the dual-luciferase reporter assay was conducted and results showed that the signal of RPSA-3′-UTR-WT+587 was significantly reduced by 44% compared with the control RPSA-3′-UTR-WT + NC (*p* < 0.001), which suggested the specific interaction between *miR-587* and the mRNA 3′-UTR of *RPSA* (Figures 4(d) and 4(e)).

**Figure 5:**
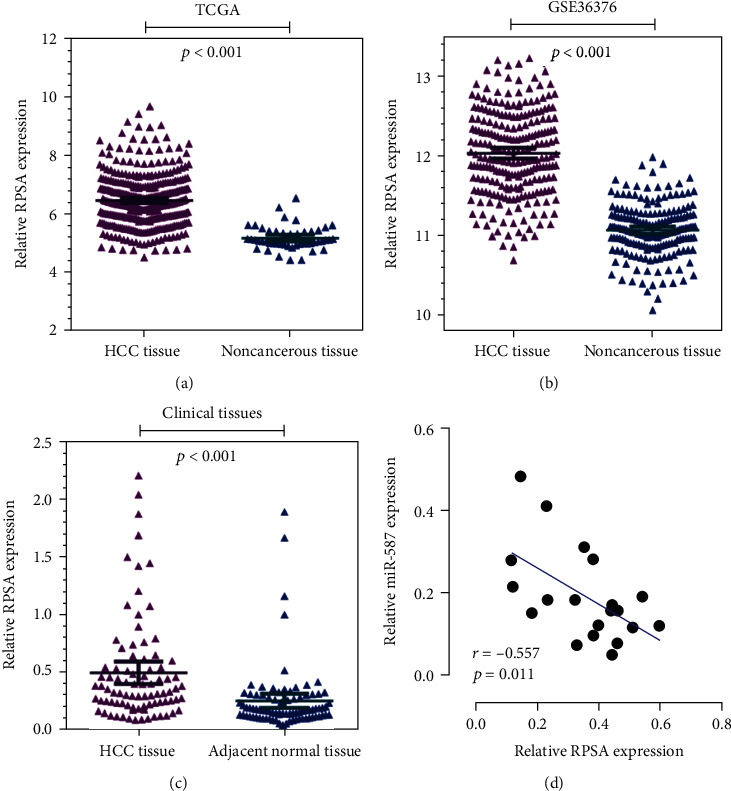
The negative regulation between *miR-587* and RPSA in HCC. The gene expression analysis based on the (a) TCGA database, (b) GEO database (GSE36376), and (c) data from 86 paired HCC tissues showed that RPSA was overexpressed in HCC tissues compared with noncancerous tissues (all *p* < 0.001). (d) Pearson's analysis uncovered a negative correlation between *miR-587* and *RPSA* expressions in HCC tissues (*p* = 0.011). miR: microRNA.

To further verify the negative regulation between *miR-587* and *RPSA*, we surveyed the TCGA and GEO (GSE36376) databases over the expression of *RPSA*, and the elevated expression of *RPSA* was identified in HCC (*p* < 0.001) (Figures 5(a) and 5(b)). The qRT-PCR analysis of 86 paired clinical tissues confirmed that *RPSA* was significantly upregulated in HCC tissues compared with the adjacent noncancerous tissues (*p* < 0.001) (Figure 5(c)). Table 1 summarizes the associations between *RPSA* expression and clinicopathologic features of HCC patients. The results showed that *RPSA* was associated with alcoholism (*p* = 0.047) and early postoperative recurrence (*p* = 0.021).

The relationship between *miR-587* and *RPSA* was further assessed between the intersection of 20 clinic HCC tissues, and the results showed a significantly negative correlation (*r* = −0.557, *p* = 0.011, Figure 5(d)). These results accurately showed that *RPSA* was a direct target of *miR-587*, and it was negatively regulated by *miR-587*.

## 5. Discussion

Identifying the vital molecules in the tumorigenesis and progression of HCC is the prerequisite for a better treatment. Recently, numerous studies have focused on the mechanisms of how microRNAs participate in the process of HCC. Dysregulated miRNAs as oncogenes or tumor suppressors are proved to be involved in the cancer-related pathways in HCC [19–21]. The *miR-199* family has emerged as tumor suppressors in HCC by targeting critical genes of MET, transmembrane glycoprotein, and mammalian target of rapamycin (mTOR) pathways [22]. *miR-221* exacerbates HCC progression by facilitating the proliferation, migration, and invasion capability of HCC cells [23]. *miR-21*, the well-characterized oncogenic miRNA, regulates the downstream genes in hypoxia, inflammation, and TET/PTEN pathways [24]. Recent studies have revealed that miRNAs including the *miR-200* family regulate the metastasis by enhancing E-cadherin expression and inhibiting EMT [25]. *miR-155* fosters cell migration and invasion in HCC cells via targeting the Ras homolog gene family member A (RhoA) [26].

Our study demonstrates that *miR-587* is downregulated in HCC tissues, revealing a tumor-suppressor role in HCC, despite its impacts on the progression and aggressiveness of the HCC cell line. Our results indicate that *miR-587* inhibits the proliferation of HCC cells through cell-cycle arrest in G1 phase and the inhibition on cell division. Zhang et al. [11] show that *miR-587*-induced suppression of PPP2R1B triggers AKT activation (the downstream) to deliver an antiapoptotic signal, which weakens the efficiency of 5-FU-induced treatment and thereby brings about drug resistance in colorectal cancer cells. However, biofunctions of *miR-587* in other cancers have not been fully explored. Herein, according to results of the dual-luciferase assay, we speculate that *miR-587* may be involved in HCC progression by targeting *RPSA*—a well-established gene in cancers. Notably, *miR-587* was downregulated, and *RPSA* was overexpressed in the HCC tissues, and the negative correlation was then confirmed. The results of the association between *miR-587*/*RPSA* expression and the clinicopathological features of HCC patients showed that low expression of *miR-587* had a higher ratio in HCC patients with MVI, and high expression of *RPSA* had a higher ratio in HCC patients with early postoperative recurrence. The early postoperative recurrence rate after HCC resection is up to 30%, which is a major threat to the poor prognosis of HCC patients [27]. MVI is a pathological feature that can only be diagnosed by postoperative histological examination. MVI has been found to increase the risk of tumor recurrence by a factor of 4.4, which was also strongly associated with aggressive tumor behavior and poor prognosis [28]. These indicate that *miR-587*/*RPSA* may be considered as clinic biomarkers for the occurrence of HCC patients.

*RPSA* gene, located on the short arm of chromosome 3, consists of 1700 base pairs and encodes 295-amino-acid proteins. It has been found that RPSA protein as an important component of 40s ribosome subunits is necessary for cell survival [15]. Like many ribosomal proteins, RPSA has a variety of functions *in vitro*, most notably as a nonintegrin cell surface layer protein-1 receptor with high affinity for laminin. Therefore, RPSA is also called 37/67 kDa laminin receptor/high-affinity laminin receptor (LRP/LR) [15]. Numerous studies have elucidated that RPSA plays a pivotal role in tumorigenesis and cancer progression in breast cancer [17], esophageal cancer [29], Alzheimer's disease [30], colorectal cancer [31], and malignant melanoma [16]. RPSA has also been implicated in the metastasis and invasion in hematological malignancies, as well as apoptosis and cellular proliferation in tumor cells [16, 32–35]. Upon laminin engaging, RPSA promotes the production of extracellular matrix and releases of laminin-derived motility fragments [36]. All this ultimately facilitates tumor cell invasion and migration. RPSA positively regulates expressions of UPA and MMP-9 in gastric cancer, which is vital for the tumor matrix and basement membrane [37]. It is characterized that RPSA is a prominent factor for cell-laminin interaction in several signaling pathways. Li et al. [38] report that RPSA activates c-Myc via the MAPK-ERK pathway, leading to an increase of FASL expression in cholangiocarcinoma cells. The inhibition of 37LRP, a precursor of RPSA, represses glioma growth and invasion, and the resulting downregulation of RPSA suppresses p-ERK1/2 and p-p38 in U251 cells, suggesting the inhibitory effect of RPSA on glioma tumorigenesis [39]. Moreover, another study reports that RPSA acts as a H_2_O_2_ sensor in H_2_O_2_-dependent modulation of cell adhesion. RPSA oxidation not only enhances the cell adhesion efficiency to laminins but also promotes cell extravasation *in vivo*. The upregulation of RPSA has been found in both primary tumors and metastatic sites, together with elevated levels of H_2_O_2_ [40]. Our results highlight that *miR-587* inhibits the proliferation, migration, and invasion of HCC cells by directly upregulating *RPSA*. However, the mechanisms of how RPSA participates in the progression of HCC have not fully unveiled. It has been proven that the level of LRP/LR is overexpressed on the membrane of liver cancer cell line HUH-7 cells on account of its interaction with laminin-1 [41]. Laminin-1 in the basement membrane of cells is found to promote cell adhesion, invasion, differentiation, growth, and migration in tumourigenic cells and biological processes [17, 42, 43]. The LRP/LR-laminin-1 interaction increases the sensitivity of proteolytic enzyme and hydrolyzes the collagen of basement membrane, leading to the degradation of basement membrane for the benefit of tumor cell invasion [44]. Moreover, the LRP/LR-laminin-1 interaction may promote tumor angiogenesis by delivering oxygen and nutrients to cancer cells [45]. These indicate that upregulated RPSA has the potential to be developed as a molecular marker for HCC. RPSA may promote the progression and aggressiveness of HCC cells through the LRP/LR-laminin-1 interaction. In this study, we for the first time report that the downexpression of *miR-587* accelerates the process of HCC by targeting the mRNA *RPSA* in HCC. However, further experiments should be conducted to address some or all of the following: biofunctions of RPSA in HCC as reported in relevant literature needs to be confirmed; moreover, the conclusion drawn from *in vitro* experiments needs to be verified in *in vivo* experiments.

In summary, *miR-587* overexpression inhibits tumor cell proliferation, migration, and invasion by directly downregulating *RPSA* mRNA in HCC. The *miR-587*/*RPSA* can be considered as a clinic biomarker for the occurrence of HCC.

## Figures and Tables

**Table 1:** Relationship between miR-587/RPSA expressions and clinicopathologic features of HCC patients.

Clinicopathological	miR-587			Patients	RPSA			Patients
	Low	High	*p* value		Low	High	*p* value	
Features	(*n* = 43)	(*n* = 43)		(*n* = 86)	(*n* = 43)	(*n* = 43)		(*n* = 86)
Gender			0.534				0.132	
Female	7	5		12	9	4		13
Male	36	38		74	34	39		73
Age(years)			0.245				0.357	
≤55	32	27		59	27	31		58
>55	11	16		27	16	12		28
Smoking status			0.808				0.194	
Negative	31	32		64	36	31		67
Positive	12	11		22	7	12		19
Alcoholism			0.795				0.047	
Negative	34	33		67	39	32		71
Positive	9	10		19	4	11		15
Size (cm)			0.223				0.194	
≤5	14	9		23	12	7		19
>5	29	34		63	31	36		67
Number			0.645				0.516	
≤2	28	30		58	22	25		47
>2	15	13		28	21	18		39
HBV			0.802				0.336	
Negative	11	10		21	10	14		24
Positive	32	33		65	33	29		62
AFP (ng/ml)			0.002				0.272	
≤400	25	11		35	20	15		35
>400	18	32		51	23	28		51
Liver cirrhosis			0.058				0.534	
No	5	12		17	7	5		12
Yes	38	31		69	36	38		74
Ascites			0.725				0.501	
No	39	38		77	37	39		76
Yes	4	5		9	6	4		10
BCLC stage			0.828				0.387	
0 + A	24	25		48	22	18		40
B + C	19	18		38	21	25		46
TNM stage			0.996				0.308	
I-II	21	20		41	22	16		38
III-IV	22	21		43	21	24		45
Distant metastasis			0.062				0.501	
Absent	34	40		74	39	37		76
Present	9	13		22	4	6		10
Tumor thrombus			0.826				0.110	
No	25	26		51	32	25		57
Yes	18	17		35	11	18		29
Microvascular cancer thrombus			0.041				0.190	
No	29	37		66	15	21		36
Yes	14	6		20	28	22		50
Early recurrence (≤12 months)			0.235				0.021	
No	28	33		61	34	24		68
Yes	15	10		25	9	19		18

miR: microRNA; AFP: alpha-fetoprotein; BCLC stage: Barcelona Clinic Liver Cancer stage.

## Data Availability

The data used to support the findings of this study are available from the corresponding author upon request.
